# Amphetamine and the Smart Drug 3,4-Methylenedioxypyrovalerone (MDPV) Induce Generalization of Fear Memory in Rats

**DOI:** 10.3389/fnmol.2019.00292

**Published:** 2019-11-29

**Authors:** Paola Colucci, Giulia Federica Mancini, Alessia Santori, Clemens Zwergel, Antonello Mai, Viviana Trezza, Benno Roozendaal, Patrizia Campolongo

**Affiliations:** ^1^Department of Physiology and Pharmacology, Sapienza University of Rome, Rome, Italy; ^2^Neurobiology of Behavior Laboratory, Santa Lucia Foundation, Rome, Italy; ^3^Department of Drug Chemistry & Technologies, Sapienza University of Rome, Rome, Italy; ^4^Department of Medicine of Precision, University of Campania Luigi Vanvitelli, Naples, Italy; ^5^Department of Science, Section of Biomedical Sciences and Technologies, University Roma Tre, Rome, Italy; ^6^Department of Cognitive Neuroscience, Radboud University Medical Center, Nijmegen, Netherlands; ^7^Donders Institute for Brain, Cognition and Behavior, Radboud University, Nijmegen, Netherlands

**Keywords:** memory accuracy, rat, behavior, inhibitory avoidance discrimination task, norepinephrine, dopamine

## Abstract

Human studies have consistently shown that drugs of abuse affect memory function. The psychostimulants amphetamine and the “bath salt” 3,4-methylenedioxypyrovalerone (MDPV) increase brain monoamine levels through a similar, yet not identical, mechanism of action. Findings indicate that amphetamine enhances the consolidation of memory for emotional experiences, but still MDPV effects on memory function are underinvestigated. Here, we tested the effects induced by these two drugs on generalization of fear memory and their relative neurobiological underpinnings. To this aim, we used a modified version of the classical inhibitory avoidance task, termed *inhibitory avoidance discrimination* task. According to such procedure, adult male Sprague–Dawley rats were first exposed to one inhibitory avoidance apparatus and, with a 1-min delay, to a second apparatus where they received an inescapable footshock. Forty-eight hours later, retention latencies were tested, in a randomized order, in the two training apparatuses as well as in a novel contextually modified apparatus to assess both strength and generalization of memory. Our results indicated that both amphetamine and MDPV induced generalization of fear memory, whereas only amphetamine enhanced memory strength. Co-administration of the β-adrenoceptor antagonist propranolol prevented the effects of both amphetamine and MDPV on the strength and generalization of memory. The dopaminergic receptor blocker cis-flupenthixol selectively reversed the amphetamine effect on memory generalization. These findings indicate that amphetamine and MDPV induce generalization of fear memory through different modulations of noradrenergic and dopaminergic neurotransmission.

## Introduction

Drugs of abuse are characterized by rewarding effects induced by the engagement of specific pathways in the brain (McHugh and Kneeland, 2019). Such rewarding effects are the principal reason that moves people to a compulsive use of these substances, which frequently ends with drug dependence (Koob, 2017). It has long been observed in humans that the intake of drugs of abuse affects memory processes (Goodman and Packard, 2016; Kutlu and Gould, 2016). More specific studies conducted in laboratory animals have been focused on which neurobiological and biochemical pathways are exploited by drugs of abuse to influence memory. Amphetamine, one of the most well-known psychostimulants, has been shown to enhance the consolidation of memory processing in rodents (McGaugh, 1973; Martinez et al., 1980a,b; Roozendaal et al., 1996; McGaugh and Roozendaal, 2009). We recently demonstrated that the 3,4-methylenedioxypyrovalerone (MDPV), a newer synthetic cathinone also known as “bath salt,” enhances short-term spatial and recognition memory performance (Atehortua-Martinez et al., 2019). Moreover, it has been shown that MDPV induces a disruption of functional connectivity networks (i.e., striatum) involved in cognitive processes (Colon-Perez et al., 2016). This new psychostimulant has recently emerged in the illegal market as a smart drug and it rapidly became highly popular (Prosser and Nelson, 2012; Baumann et al., 2017). However, its fame is also associated with several important adverse effects, and among these, long-term cognitive impairments in humans have been documented (Karila et al., 2015). One *in vitro* study on MDPV activity demonstrated that it has a similar, yet not identical, mechanism of action compared to amphetamine. Indeed, both drugs of abuse have the same molecular targets represented by the norepinephrine (NE), dopamine (DA) and serotonin re-uptake transporters (NET, DAT and SERT, respectively), but MDPV displays greater potency than amphetamine with regard to DA re-uptake transport (Baumann et al., 2013). Amphetamine effects on memory consolidation are dependent on its pharmacological action which increases NE and DA release (Martinez et al., 1983; LaLumiere et al., 2005; Fleckenstein et al., 2007; Roozendaal et al., 2008). Very recently, it has been shown that the effect on short-term memory induced by MDPV is linked to D1 dopaminergic receptor activation (Atehortua-Martinez et al., 2019). The role of noradrenergic and dopaminergic neurotransmission on memory, especially for the consolidation phase, is well established (LaLumiere et al., 2005; Roozendaal et al., 2008; Schwabe, 2017; Quaedflieg and Schwabe, 2018; Wideman et al., 2018). Although it has been demonstrated that both amphetamine and MDPV can affect memory retention, no evidence exists on whether such drugs can also affect the quality of memory. The study about the influence of drugs of abuse on the quality of memory increasingly acquired attention during the last century and is just nowadays growingly becoming an intriguing issue, even if up to date there are only sparse studies (Easton and Bauer, 1997; Koriat et al., 2000; Loftus, 2005; Ballard et al., 2012; Oeberst and Blank, 2012; Carter et al., 2013; Horry et al., 2014; Hoscheidt et al., 2014). However, the study of the mechanisms through which drugs of abuse affect memory quality could be a riveting topic, mainly in the light of increasing evidence that drugs of abuse (e.g., psychedelic drugs, hallucinogens) can alter the experience of reality (Bøhling, 2017). Such altered perception might be one of the causes why some people are prompted to a recreational use of such substances (Kjellgren and Soussan, 2011; Móró et al., 2011), thus making it an important and urgent issue to be investigated. Emotions have a considerable impact on memory (Tyng et al., 2017), for example, when an aversive stimulus occurs, the associated fear leads to remember the information over time (Rogan et al., 1997), but sometimes the accuracy of such emotional memory can be altered and distorted over time, eventually leading to memory generalization (Asok et al., 2018). This emotional/fear generalization effect has been studied for many decades through the contextual fear conditioning paradigm (Rohrbaugh and Riccio, 1968; Ruediger et al., 2011). Recently, a novel experimental model suitable to investigate both strength and accuracy of memory has been validated for rodents (Atucha and Roozendaal, 2015; Atucha et al., 2017): the inhibitory avoidance discrimination task. This task allows to evaluate whether fear memory associated with footshock can be generalized to a novel and safe, yet similar context. Hence, the aim of the present study was to investigate whether the two psychostimulants amphetamine and MDPV affect generalization of fear memory to a novel and safe yet similar context using an inhibitory avoidance discrimination task. Since both amphetamine and MDPV modulate NE and DA tone, we also aimed at evaluating the involvement of the noradrenergic and dopaminergic systems in mediating the effects of amphetamine and MDPV on fear memory generalization.

## Materials and Methods

### Animals and Procedures

Male adult Sprague–Dawley rats (320–370 g at the time of behavioral experiments) from Charles River Laboratories (Calco, Italy) were housed individually in a temperature-controlled (21 ± 1°C) vivarium room and maintained under a 12 h/12 h light/dark cycle (7:00 A.M. to 7:00 P.M. lights on). Food and water were available *ad libitum*. Rats were handled for 1 min for three consecutive days prior to training. Training and testing were performed during the light phase of the cycle between 11:00 A.M. and 2:00 P.M. All procedures involving animal care or treatments were performed in compliance with the ARRIVE guidelines, Directive 2010/63/EU of the European Parliament, the D. L. 26/2014 of the Italian Ministry of Health, the Declaration of Helsinki and the Guide for the Care and Use of Mammals in Neuroscience and Behavioral Research (National Research Council, 2004).

### Inhibitory Avoidance Discrimination Task

For all experiments, rats were trained and tested on a modified version of the classic inhibitory avoidance task, termed inhibitory avoidance discrimination task, that allows to investigate both strength and accuracy of memory (Atucha and Roozendaal, 2015; Atucha et al., 2017). Rats were subsequently trained in two contextually distinct inhibitory avoidance apparatuses within a single training session, but footshock was delivered only in the latter context. On the retention test, all animals were tested in both training contexts as well as in a novel context. These training and test procedures, as previously demonstrated by Atucha and Roozendaal (2015), allow to investigate whether rats remember the two contexts they visited during the training trial, as well as if they display a specific episodic-like memory of the association between footshock and the correct training context. Each apparatus had the same geometry and consisted of a trough-shaped alley (91 cm long, 15 cm deep, 20 cm wide at the top, and 6.4 cm wide at the bottom) divided into two compartments, separated by a sliding door that opened by retracting into the floor. The starting compartment (31 cm) was made of opaque white plastic and was well lit; the shock compartment (60 cm) was made of two dark, electrifiable metal plates and was not illuminated. The training context in which footshock was given (Shock box) did not have any contextual modifications. The safe training context (Non-Shock box) had four vertical white stripes (2 cm wide) taped in the dark compartment together with tape placed on the floor, closing the gap between the two plates. The Novel box (used on the retention test only) had two white circles (3.5 cm diameter) taped on each wall of the dark compartment, and the gap between the plates was closed with tape. All three inhibitory avoidance apparatuses were located next to one another in a sound- and light-attenuated room.

**Figure 1:**
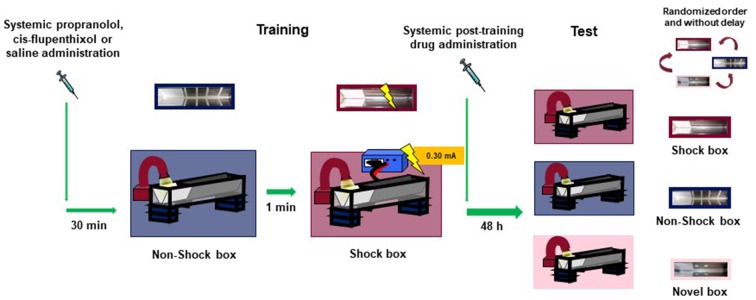
Schematic representation of the experimental design.

For training, rats were initially placed in the starting compartment of the Non-Shock box and their latency to enter the dark compartment with all four paws (maximum latency of 30 s) was recorded. No footshock was delivered in this box. Afterward, the rats were removed from the apparatus and, after a delay of 1-min, placed in the starting compartment of the second inhibitory avoidance apparatus (Shock box). We selected a 1-min delay because, as previously demonstrated (Atucha and Roozendaal, 2015), although animals do not discriminate between the two training contexts with such short interval between the two training episodes, the fear does not generalize to a novel context. After the rat stepped completely into the dark compartment, the sliding door was closed and a single inescapable footshock (0.30 mA; 1 s) was delivered. Rats were removed from the apparatus 20 s after termination of footshock and, after drug treatment, returned to their home cage. On the retention test, 2 days after training, all animals were tested, in a randomized order and without delay, in the two training contexts (i.e., Shock box and Non-Shock box) and in a Novel box they had not visited before. No footshock was delivered on the retention test trial, and for all three boxes, rats were placed in the starting compartment and their latency to enter the dark compartment with all four paws (maximum latency of 600 s) was recorded. Longer latencies in the Shock box compared with the Non-Shock or Novel box were interpreted as indicating accurate memory of the shock–context association. Moreover, long retention latencies in all the three boxes were considered as an index of memory generalization across contexts. Immediately after the training or testing of each animal, each apparatus was wiped clean with a 70% ethanol solution. The experimental design is illustrated in Figure 1.

### Drug Administration

Amphetamine [(RS)-1-phenylpropan-2-amine; 1 and 3 mg/kg] and 3,4-methylenedioxypyrovalerone (MDPV; 0.5 and 1 mg/kg) were dissolved in saline (vehicle) and administered intraperitoneally, at the volume of 1 ml/kg, immediately after the training session (Figure 1). In the second experiment, to examine whether the amphetamine and MDPV effects on memory involve the noradrenergic system, the β-adrenoceptor antagonist propranolol (1-naphthalen-1-yloxy-3-propan-2-ylaminopropan-2-ol; 1 mg/kg) or saline (vehicle) was administered intraperitoneally 30 min prior to training, followed by amphetamine (3 mg/kg), MDPV (1 mg/kg) or saline immediately after training (Figure 1). In the third experiment, to investigate the involvement of the dopaminergic system in mediating amphetamine and MDPV effects on memory, the non-selective D1/D2 dopaminergic receptor antagonist cis-flupenthixol (2-[4-[(3Z)-3-[2-(trifluoromethyl)thioxanthen-9-ylidene]propyl]piperazin-1-yl]ethanol; 0.25 mg/kg) or saline (vehicle) was administered intraperitoneally 30 min prior to training, followed by an immediate post-training intraperitoneal injection of amphetamine (3 mg/kg), MDPV (1 mg/kg) or saline (Figure 1). Drug doses were chosen on the basis of literature data (Roozendaal et al., 2004; Trost and Hauber, 2014) also showing that MPDV has a greater pharmacological potency than amphetamine (Baumann et al., 2013). All drugs were dissolved in sterile 0.9% saline. Drug solutions were freshly prepared before each experiment.

### Statistical Analysis

Data are expressed as mean ± SEM. All data were analyzed with ANOVA for Repeated Measures (RM ANOVA) with drug treatment as between-group factor and retention latencies of individual animals in the different test contexts (Shock, Non-Shock, and Novel boxes) as repeated measure. Two-way ANOVAs were used to analyze retention latencies of rats treated with propranolol vs. saline alone and cis-flupenthixol vs. saline alone. The source of the detected significances was determined by Tukey–Kramer *post hoc* tests for between and within-group differences. *P*-values of less than 0.05 were considered statistically significant. The number of rats per group is indicated in the figure legends.

## Results

### Amphetamine and MDPV Induce Memory Generalization in an Inhibitory Avoidance Discrimination Task

**Figure 2:**
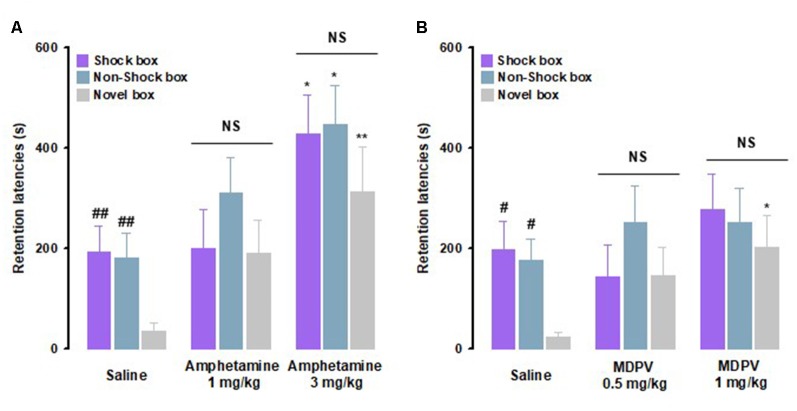
Amphetamine and 3,4-methylenedioxypyrovalerone (MDPV) induce memory generalization of inhibitory avoidance discrimination task. On the 48-h retention test, rats were sequentially tested in all three contextually modified inhibitory avoidance apparatuses in a random order and their retention latencies were analyzed. **(A)** Retention latencies of amphetamine and saline-treated rats. Saline-treated animals showed longer retention latencies in the Shock box and Non-Shock box compared to those induced in the Novel box. In all three boxes, amphetamine 3 mg/kg induced higher retention latencies than saline-treated rats. ^##^*P* < 0.01 saline group latencies in the Shock box or Non-Shock box vs. saline group latencies in the Novel box; *P < 0.05, **P < 0.01 amphetamine 3 mg/kg latencies in the Shock box, Non-Shock box or Novel box vs. saline group in the Shock box, Non-Shock box or Novel box; NS, no significant differences (*n* = 9–13 rats). **(B)** Retention latencies of MDPV and saline-treated rats. Saline-treated animals showed longer retention latencies in the Shock box and Non-Shock box compared to those induced in the Novel box. In the Novel box retention latencies induced by MDPV 1 mg/kg were significantly longer than those induced by saline-treated rats in the same box. ^#^*P* < 0.05 saline group latencies in the Shock box or Non-Shock box vs. saline group latencies in the Novel box; *P < 0.05 MDPV 1 mg/kg treated group latencies in the Novel box vs. saline group latencies in the Novel box; NS, no significant differences (*n* = 10–12 rats).

Rats were trained on the inhibitory avoidance discrimination task and given an immediate post-training intraperitoneal injection of amphetamine, MDPV or saline. With regard to amphetamine effects, as shown in Figure 2A, RM ANOVA for retention latencies indicated significant effects for treatment (*F*_(2,29)_ = 10.23, *P* < 0.01) as well as context (*F*_(2,29)_ = 4.08, *P* = 0.02), but no significant interaction between these two factors (*F*_(4,58)_ = 0.48, *P* = 0.75). *Post hoc* analysis, in accordance to what it has been previously demonstrated (Atucha and Roozendaal, 2015), revealed that saline-treated animals showed longer retention latencies in the Shock box (*P* < 0.01) and Non-Shock box (*P* < 0.01) compared to those in the Novel box, indicating that saline-treated rats were able to discriminate the two training contexts from the new one they had visited only during the test trial (Figure 2A). Retention latencies in the Shock box of rats treated with amphetamine (3 mg/kg) were significantly longer than those of animals treated with saline (*P* < 0.05), indicating that amphetamine, at the higher dose tested, enhanced the strength of memory. Furthermore, amphetamine (3 mg/kg)-treated rats showed longer retention latencies in both the Non-Shock box (*P* < 0.05) and Novel box (*P* < 0.01) compared to saline-treated animals. Thus, these results revealed that amphetamine-induced memory generalization across contexts.

With regard to MDPV effects, as shown in Figure 2B, RM ANOVA for retention latencies indicated no significant effect for treatment (*F*_(2,30)_ = 1.83, *P* = 0.18), a significant context effect (*F*_(2,30)_ = 3.37, *P* = 0.04), and no significant interaction between these two factors (*F*_(2,60)_ = 1.04, *P* = 0.39). *Post hoc* analysis confirmed that the performance of control animals was the same as for the amphetamine experiments (Figure 2B). Retention latencies of animals treated with MDPV (1 mg/kg) did not differ from those of saline-treated controls in both Shock and Non-Shock boxes but were significantly longer than those of saline-treated animals (*P* < 0.05) in the Novel box. These results show that rats that were treated with MDPV (1 mg/kg) had similar retention latencies in all three boxes, indicating that MDPV induced generalization across contexts. Taken together, these findings indicate that amphetamine and MDPV have differential effects on memory strength, but that both drugs increase generalization of fear memory to a novel safe context.

All training latencies are shown in Supplementary Table S1.

### Noradrenergic System Activation Mediates the Effects of Amphetamine and MDPV on Memory Generalization

We sought to test whether the amphetamine- and MDPV-mediated effects on strength and generalization of memory involved activation of the noradrenergic system. Herein, rats were given intraperitoneal injections of the β-adrenoceptor antagonist propranolol or saline 30 min prior to training, followed by post-training administrations of the effective doses of amphetamine (3 mg/kg), MDPV (1 mg/kg), or their corresponding vehicles.

**Figure 3:**
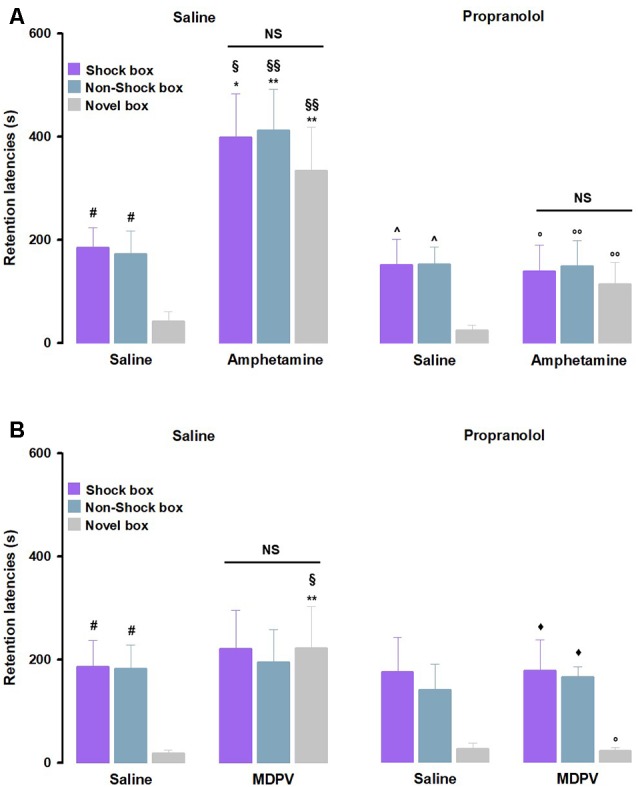
Noradrenergic activation mediates amphetamine and MDPV effects on memory generalization. On the 48-h retention test, rats were sequentially tested in all three contextually modified inhibitory avoidance apparatuses in a random order and their retention latencies were analyzed. **(A)** Retention latencies of rats treated with propranolol or saline 30 min prior to training together with amphetamine or saline administered immediately after training. Saline alone-treated animals showed longer retention latencies in the Shock box and Non-Shock box compared to those induced in the Novel box, the same happens for the propranolol alone-treated animals. In all three boxes, amphetamine alone-treated rats showed higher retention latencies than saline alone-treated rats and then those exerted by rats given propranolol alone. Retention latencies of the group treated with propranolol together with amphetamine in all three boxes were significantly lower compared to those of amphetamine alone-treated rats. ^#^*P* < 0.05 saline group latencies in the Shock box or Non-Shock box vs. saline group latencies in the Novel box; ^∧^*P* < 0.05 propranolol alone latencies in the Shock box or Non-Shock box vs. propranolol alone latencies in the Novel box; *P < 0.05, **P < 0.01 amphetamine alone-treated group latencies in the Shock box, Non-Shock box or Novel box vs. saline group latencies in the Shock box, Non-Shock box or Novel box; ^§^*P* < 0.05, ^§§^*P* < 0.01 amphetamine alone-treated group latencies in the Shock box, Non-Shock box or Novel box vs. propranolol alone group latencies in the Shock box, Non-Shock box or Novel box; °*P* < 0.05, ^°°^*P* < 0.01 propranolol and amphetamine-treated group latencies in the Shock box, Non-Shock box or Novel box vs. amphetamine alone-treated group latencies in the Shock box, Non-Shock box or Novel box; NS, no significant differences (*n* = 9–13 rats). **(B)** Retention latencies of rats treated with propranolol or saline 30 min prior to training together with MDPV or saline administered immediately after training. Saline alone-treated animals showed longer retention latencies in the Shock box and Non-Shock box compared to those induced in the Novel box, the same happens for the propranolol together with MDPV-treated animals. In the Novel box retention latencies induced by MDPV alone treatment were significantly longer than those exerted by rats treated with saline alone and propranolol alone. Retention latencies of the group treated with propranolol together with MDPV in the Novel box were significantly lower compared to those of MDPV alone-treated rats. ^#^*P* < 0.05 saline group latencies in the Shock box or Non-Shock box vs. saline group latencies in the Novel box; ^⧫^*P* < 0.05 propranolol together with MDPV latencies in the Shock box or Non-Shock box vs. propranolol together with MDPV latencies in the Novel box; **P < 0.01, MDPV alone-treated group latencies in the Novel box vs. saline group latencies in the Novel box; ^§^*P* < 0.05, MDPV alone-treated group latencies in the Novel box vs. propranolol alone-treated group latencies in the Novel box; °*P* < 0.05, propranolol and MDPV-treated group latencies in the Novel box vs. MDPV alone-treated group in the Novel box; NS, no significant differences (*n* = 8–11 rats).

To investigate whether the noradrenergic system influences on amphetamine-mediated effects on memory generalization, we first analyzed retention latencies of saline- and propranolol alone-treated animals in the three contexts (Figure 3A). RM ANOVA for retention latencies of the saline-treated animals showed a significant effect of context (*F*_(2,36)_ = 4.80, *P* = 0.01). Similar to the control rats described above, *post hoc* analysis confirmed that saline-treated animals showed longer retention latencies in the Shock box (*P* < 0.05) and Non-Shock box (*P* < 0.05) as compared to those in the Novel box, thus indicating that control rats were able to discriminate the two training contexts from the new one that they visited only during the test trial. The same results were obtained with the RM ANOVA analysis for retention latencies of propranolol alone-treated animals (*F*_(2,35)_ = 4.52, *P* = 0.02). *Post hoc* analysis revealed that propranolol alone-treated rats showed longer retention latencies in the Shock box (*P* < 0.05) and Non-Shock box (*P* < 0.05) as compared to those in the Novel box. These findings indicate that also rats that were treated with propranolol accurately remembered the two training contexts, even if they were not able to discriminate in which training context they received the footshock. Moreover, two-way ANOVA for retention latencies of rats treated with saline and propranolol did not reveal a significant treatment effect (*F*_(1,69)_ = 0.59, *P* = 0.44) or treatment × context interaction effect (*F*_(2,69)_ = 0.03, *P* = 0.97), but revealed a significant effect of the context (*F*_(2,69)_ = 9.23, *P* < 0.0001), suggesting that treatment does not affect animals memory retention for different apparatuses (Figure 3A). As shown in Figure 3A, as for the noradrenergic influences in the amphetamine effects on memory function, RM-ANOVA for retention latencies revealed significant effects of treatment (*F*_(3,42)_ = 11.70, *P* < 0.01) as well as context (*F*_(2,42)_ = 6.01, *P* < 0.01), and no significant differences for the interaction between both factors (*F*_(6,84)_ = 0.50, *P* = 0.80). Retention latencies of rats treated with amphetamine alone in the Shock box (*P* < 0.05), Non-Shock box (*P* < 0.05) and Novel box (*P* < 0.01) were all significantly longer than those displayed by saline-treated animals in the same boxes. Retention latencies of rats that were treated with propranolol together with amphetamine in the Shock box (*P* < 0.05), Non-Shock box (*P* < 0.01) and Novel box (*P* < 0.01) were significantly shorter compared to those of animals treated with amphetamine alone in the same boxes. Moreover, retention latencies of rats treated with amphetamine alone in the Shock box (*P* < 0.05), Non-Shock box (*P* < 0.01) and Novel box (*P* < 0.01) were significantly longer than those of rats treated with propranolol alone in the same boxes.

To evaluate whether noradrenergic activity is also involved in the modulation of the MDPV effects on memory generalization, we analyzed retention latencies of both saline and propranolol alone-treated animals and confirmed the results that we described above for the experiments involving amphetamine (Figure 3B). Furthermore, as previously described, also in this experiment no significant differences were found between saline and propranolol alone-treated rats (Figure 3B).

As shown in Figure 3B, RM ANOVA for retention latencies indicated no significant effect of treatment (*F*_(3,32)_ = 1.70, *P* = 0.19) or treatment × context interaction effect (*F*_(6,64)_ = 1.12, *P* = 0.36), but revealed a significant effect of the context (*F*_(2,32)_ = 7.32, *P* < 0.01). Rats treated with MDPV alone showed longer retention latencies in the Novel box than those of saline alone- (*P* < 0.01) or propranolol alone-treated rats (*P* < 0.05) exposed to the same box. Moreover, retention latencies of animals treated with propranolol together with MDPV in the Shock-box were significantly longer compared to the Novel box (*P* < 0.05) and in the Non-Shock box compared to the Novel box (*P* < 0.05). Particularly in the Novel box, retention latencies of animals treated with propranolol together with MDPV were significantly shorter compared to those of MDPV alone-treated animals in the same box.

In summary, these findings indicate that the amphetamine effect on enhancing memory strength is mediated by the noradrenergic system. Moreover, our findings indicate that the amphetamine effect on memory generalization appears to be only partially due to a modulation of the noradrenergic system, whereas the memory generalization effect induced by MDPV is entirely dependent on noradrenergic activity.

All training latencies are indicated in Supplementary Table S2.

### Dopaminergic System Activation Mediates the Effects of Amphetamine, but Not MDPV, on Memory Generalization

In this set of experiments, we tested whether dopaminergic activity is involved in the effects induced by amphetamine and MDPV on memory generalization. To this aim, rats were intraperitoneally treated with the DA receptor antagonist cis-flupenthixol or saline 30 min before the training trial and subjected to post-training administration of the effective doses of amphetamine (3 mg/kg), MDPV (1 mg/kg), or their corresponding vehicle solutions.

**Figure 4:**
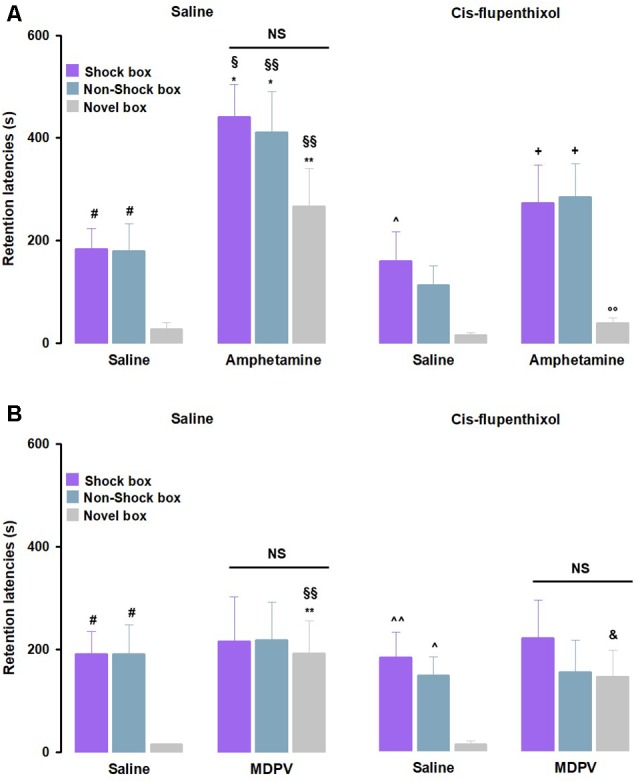
Dopaminergic activation mediates the effects induced by amphetamine, but not MDPV, on memory generalization. On the 48-h retention test, rats were sequentially tested in all three contextually modified inhibitory avoidance apparatuses in a random order and their retention latencies were analyzed. **(A)** Retention latencies of rats treated with cis-flupenthixol or saline 30 min prior to training together with amphetamine or saline administered immediately after training. Saline alone-treated animals showed longer retention latencies in the Shock box and Non-Shock box compared to those induced in the Novel box. Cis-flupenthixol alone-treated animals showed higher retention latencies in Shock box compared only to those showed in the Novel box. Cis-flupenthixol together with amphetamine treated-rats showed longer retention latencies in the Shock box and Non-Shock box compared to those induced in the Novel box. In all three boxes, amphetamine alone-treated rats showed higher retention latencies than saline alone-treated rats and cis-flupenthixol alone-treated rats. Retention latencies of rats treated with cis-flupenthixol together with amphetamine were significantly lower than those of amphetamine alone-treated rats, only in the Novel box. ^#^*P* < 0.05 saline group latencies in the Shock box or Non-Shock box vs. saline group latencies in the Novel box; ^∧^*P* < 0.05 cis-flupenthixol alone latencies in the Shock box vs. cis-flupenthixol alone latencies in the Novel box; ^+^*P* < 0.05, cis-flupenthixol together with amphetamine latencies in the Shock or Non-Shock box vs. cis-flupenthixol together with amphetamine latencies in the Novel box; *P < 0.05, **P < 0.01, amphetamine alone-treated group latencies in the Shock box, Non-Shock box or Novel box vs. saline group latencies in the Shock box, Non-Shock box or Novel box; ^§^*P* < 0.05, ^§§^*P* < 0.01, amphetamine alone group latencies in the Shock box, Non-Shock box or Novel box vs. cis-flupenthixol alone-treated group latencies in the Shock box, Non-Shock box or Novel box; ^°°^*P* < 0.01, cis-flupenthixol and amphetamine-treated group latencies in the Novel box vs. amphetamine alone-treated group in the Novel box; NS, no significant differences (*n* = 9–10 rats). **(B)** Retention latencies of rats treated with cis-flupenthixol or saline 30 min prior to training together with MDPV or saline administered immediately after training. Saline alone-treated animals showed longer retention latencies in the Shock box and Non-Shock box compared to those induced in the Novel box, the same happens to cis-flupenthixol alone-treated animals. In the Novel box, MDPV alone-treated rats showed higher latencies with respect to saline-treated rats and cis-flupenthixol alone-treated rats; cis-flupenthixol and MDPV-treated rats showed higher latencies with respect to cis-flupenthixol alone-treated rats and with respect to cis-flupenthixol alone-treated. ^#^*P* < 0.05 saline group latencies in the Shock box or Non-Shock box vs. saline group latencies in the Novel box; ^∧^*P* < 0.05, ^∧∧^*P* < 0.01, cis-flupenthixol alone latencies in the Shock box or Non-shock box vs. cis-flupenthixol alone latencies in the Novel box; **P < 0.01, MDPV alone-treated group latencies in the Novel box vs. saline group latencies in the Novel box; ^§§^*P* < 0.01, MDPV alone-treated group latencies in the Novel box vs. cis-flupenthixol alone-treated group in the Novel box; ^&^*P* < 0.05, cis-flupenthixol together with MDPV retention latencies in the Novel box vs. cis-flupenthixol alone latencies in the Novel box; NS, no significant differences (*n* = 8–11 rats).

As previously done in the experiments involving the noradrenergic system, we first analyzed the retention latencies of saline- and of cis-flupenthixol alone-treated animals in the three experimental contexts. Animals that were treated with saline showed comparable latencies to control groups that were discussed above (Figure 4A). Moreover, in line with the previous set of experiments, no significant differences between saline- and cis-flupenthixol alone-treated animals (Figure 4A) were detected. As for the involvement of the dopaminergic system in the amphetamine effects on memory function, as shown in Figure 4A, RM ANOVA for retention latencies indicated significant effects of treatment (*F*_(3,34)_ = 10.87, *P* < 0.01) and context (*F*_(2,34)_ = 17.62, *P* < 0.01), but not significant interaction between both factors (*F*_(6,68)_ = 0.47, *P* = 0.83) effect. *Post hoc* analysis revealed that retention latencies of amphetamine alone-treated rats were significantly longer than those of rats that were given saline alone in the Shock box (*P* < 0.05), Non-Shock box (*P* < 0.05) and Novel box (*P* < 0.01). Retention latencies of rats that were treated with amphetamine alone were significantly longer than those of cis-flupenthixol alone-treated rats in the Shock box (*P* < 0.05), Non-Shock box (*P* < 0.01) and Novel box (*P* < 0.01). Retention latencies in the Novel box of rats treated with cis-flupenthixol together with amphetamine were significantly shorter with respect to rats given amphetamine alone (*P* < 0.01) in the same box. Moreover, they showed longer latencies in the Shock box and in the Non-Shock box compared to the Novel box (*P* < 0.05).

Concerning the dopaminergic role on MDPV-mediated generalization effects on memory, for the retention latencies of both saline- and cis-flupenthixol alone-treated rats, we confirmed the same results as described above (Figure 4B); again, no significant differences were found between the two treatment groups (Figure 4B). As shown in Figure 4B, RM ANOVA for retention latencies indicated no significant treatment effect (*F*_(3,38)_ = 1.71, *P* = 0.18), a significant effect of the context (*F*_(2,38)_ = 5.06, *P* < 0.01) and no significant interaction between these two factors (*F*_(6,76)_ = 0.81, *P* = 0.56) effect. *Post hoc* analysis revealed that retention latencies of rats treated with MDPV alone were significantly longer than those of rats given saline alone and cis-flupenthixol alone in the Novel box (*P* < 0.01), and that the retention latencies of rats treated with cis-flupenthixol together with MDPV were significantly longer than those of rats given saline alone and cis-flupenthixol alone in the Novel box (*P* < 0.05).

In conclusion, these results demonstrated that the dopaminergic system is involved in modulating the effects of amphetamine on memory generalization as well with only a partial interference on its effects on memory strength. However, the blockade of DA receptors does not influence MDPV effects on memory generalization.

All training latencies are shown in Supplementary Table S3.

## Discussion

The present findings indicate that amphetamine and MDPV have different effects on memory strength, but both drugs increase generalization of fear memory to a novel safe context. We further show that noradrenergic and dopaminergic neurotransmission is differentially involved in the effects mediated by amphetamine and MDPV on memory. As previously showed, saline-treated animals trained in the inhibitory avoidance discrimination task, with a 1-min interval between the two training apparatuses, were able to discriminate the two training contexts from the new one visited only during the test trial (Atucha and Roozendaal, 2015), indicating that fear memory associated with footshock did not generalize to the novel safe box. Here, we specifically selected this short time delay to evaluate whether amphetamine and MDPV could induce fear memory generalization of footshock to the novel safe context. Our findings first demonstrate, in accordance to previous reports (McGaugh, 1973; Martinez et al., 1980a,b; Roozendaal et al., 1996; McGaugh and Roozendaal, 2009), that amphetamine increases memory strength as indicated by the longer retention latencies in the Shock box. Of more interest, we also found that amphetamine induces fear memory generalization by enhancing retention latencies in all three boxes, including the box never visited before. MDPV did not directly affect memory strength, but induced generalization of memory, as well as demonstrated by the finding that MDPV-treated animals exerted similar retention latencies in all three boxes. Such evidence that both psychostimulants induce fear memory generalization to a context to which animals were never exposed before is a truly novel and important finding.

Previous studies have indicated that both amphetamine and MDPV, through a similar, yet not identical, mechanism of action increase brain monoamines release, particularly NE and DA, two neurotransmitters extensively involved in the modulation of memory (LaLumiere et al., 2005; McGaugh and Roozendaal, 2009). In fact, amphetamine acts as a substrate of NET, DAT and SERT inducing a “reverse transport” of neurotransmitters (Robertson et al., 2009), whereas MDPV, like cocaine, is an inhibitor of NET, DAT and SERT (Simmler et al., 2013; Marusich et al., 2014; Baumann et al., 2017). Amphetamine also interacts with the vesicular monoamine transporter (VMAT), in particular VMAT2, depleting synaptic vesicles of their neurotransmitter content (Teng et al., 1998; Eiden and Weihe, 2011), and inhibits monoaminooxidase (MAO), which is a family of enzymes that catalyzes monoamine oxidation (Miller et al., 1980; Liu et al., 2016). The affinity between MDPV and MAO has not yet been investigated. Literature data indicate that two other synthetic cathinones, mephedrone and methylone, have a similar mechanism of action of amphetamine but present a lower affinity for VMAT2 and probably decrease activity on MAO with respect to amphetamine (Baumann et al., 2017). There is evidence that MDPV is more powerful as an uptake blocker of DAT than of NET and SERT (Baumann et al., 2017). Therefore, although this remains purely speculative, it is possible that the different effects induced by amphetamine and MDPV on memory strength may be related to the variation of the specific expression of these monoamine transporters in different brain regions.

Notwithstanding the different mechanism of action through which these two psychostimulants enhance NE and DA levels, both drugs of abuse enhance noradrenergic and dopaminergic neurotransmission (Robertson et al., 2009; Baumann et al., 2013) and the involvement of these two systems on the effects induced by drugs of abuse on memory strength and generalization had not been previously investigated. Here, we found that noradrenergic influences, mediated by an action on β-adrenoceptors, were responsible for the enhancing effects of amphetamine on memory consolidation. Extensive evidence indicates that noradrenergic activation is crucially involved in regulating memory consolidation for emotional experiences (Gold et al., 1975; Gallagher et al., 1977; Gold and van Buskirk, 1978; Liang et al., 1986; McIntyre et al., 2003; Ferry et al., 2015; LaLumiere et al., 2017). Hence, it is possible that amphetamine effects on memory strength could be due to an indirect activation of central β-adrenoceptors. Of more novel interest, we demonstrated that the noradrenergic system also modulates the generalization effects induced by both amphetamine and MDPV. In particular, our findings indicate that amphetamine effects on generalization are partially blocked by preventive administration of the β-adrenoceptor antagonist propranolol, while MDPV effects are totally blocked. Previous findings demonstrated that the administration of the physiological noradrenergic stimulant yohimbine, a selective α2-adrenoceptor antagonist, ameliorates the accuracy of memory in the inhibitory avoidance discrimination task (Atucha and Roozendaal, 2015) and that NA infusion into the basolateral amygdala maintains accuracy of episodic-like memory of the two distinct training contexts, preventing the generalization effect induced by a memory reorganization over time (Atucha et al., 2017). However, our results unexpectedly suggest that if the noradrenergic system is activated by a drug of abuse it alters memory accuracy, inducing generalization. This effect could be explained considering the activation of the noradrenergic system in brain areas particularly involved in memory generalization, such as medial prefrontal cortex, nucleus reunions, and hippocampus (Xu and Sudhof, 2013). Conversely, no data are available with regard to the potential role of dopaminergic modulation on memory accuracy. Herein, we demonstrate that the dopaminergic system is involved in modulating the effects of amphetamine on memory generalization as well with only a partial interference on memory strength. However, the blockade of DA receptors does not influence MDPV effects on memory generalization. Together these findings indicate that the generalization effect induced by amphetamine is strongly regulated by the dopaminergic system, whereas the MDPV effects on memory generalization seem to be due to a selective activation of the noradrenergic system. Although these results require further investigation, it can be hypothesized that there is a differential recruitment induced by amphetamine and MDPV on the monoamine systems in different brain areas.

Brain regions with a high density of DAT and dopaminergic receptors, such as the striatum and nucleus accumbens (Efimova et al., 2016) may be responsible for regulating amphetamine effects on memory generalization. Conversely, it is possible that the effects of MDPV on memory generalization are linked to brain areas with high levels of NET and β-adrenoceptors such as the dentate gyrus of the hippocampus and the perirhinal cortex, which are known to play a critical role in the regulation of memory discrimination (Miranda et al., 2017; van Dijk and Fenton, 2018). In agreement with these results, it could be hypothesized that the generalization induced by MDPV is mediated by β-adrenoceptors in such brain areas. Thus, our findings demonstrate that both amphetamine and MDPV induce generalization of fear memory *via* a different involvement of NE and DA neurotransmission. These results pave the way for future studies aimed at investigating the role of specific brain areas in mediating the differential effects of both psychostimulant drugs on strength and quality of memory, thus ultimately leading to reveal the neurobiological underpinnings of memory alterations induced by drugs of abuse.

## Data Availability Statement

The datasets generated for this study are available on request to the corresponding author.

## Ethics Statement

The animal study was reviewed and approved by Italian Ministry of Health.

## Author Contributions

All authors contributed to and have approved the final manuscript. PCo and GM contributed to the design of the experiments, performed the experiments, analyzed data and wrote the manuscript. AS and CZ performed the experiments and analyzed data. AM and VT contributed to the design of the experiments. BR contributed to the design of the experiments and wrote the manuscript. PCa supervised the project, designed the experiments and wrote the manuscript. All authors read and approved the final version of the manuscript.

## Conflict of Interest

The authors declare that the research was conducted in the absence of any commercial or financial relationships that could be construed as a potential conflict of interest.
