# Supplementary material for: Amphetamine and the Smart Drug 3,4-Methylenedioxypyrovalerone (MDPV) Induce Generalization of Fear Memory in Rats
Source: Front Mol Neurosci. 2019 Nov 29;12:292. doi: 10.3389/fnmol.2019.00292 (PMC6895769; doi:10.3389/fnmol.2019.00292)
Supplement: Supplementary file 1 [file Table_1.docx]

Supplementary Material

# Amphetamine and MDPV induce memory generalization of inhibitory avoidance discrimination task

During the training trial rats were first exposed to the Non-Shock box and, with a 1-min delay, to the Shock one. Approach latencies to enter the dark compartment, before footshock, were evaluated. Immediately after the training, rats received an intraperitoneal injection of amphetamine (1 and 3 mg/kg), MDPV (0.5 and 1 mg/kg) or saline. In the group given post-training injection of amphetamine or saline, RM ANOVA for approach latencies did not reveal significant effects of post-training treatment (F(2,29) = 0.17, P = 0.85), context (F(1,29) = 1.47, P = 0.24) or interaction between these two factors (F(2,29) = 0.47, P = 0.63) (Table 1). In the group given post-training injection of MDPV or saline, RM ANOVA for approach latencies revealed no significant effects of post-training treatment (F(2,30) = 0.05, P = 0.95), context (F(1,30) = 0.32, P = 0.58) or interaction between these two factors (F(2,30) = 0.17, P = 0.84) (Table 1).

# Noradrenergic activation mediates amphetamine and MDPV effects on memory generalization

Thirty min prior to training rats were given an intraperitoneal injection of the β-adrenoceptors antagonist propranolol (1 mg/kg) or saline together with post-training administration of the effective doses of amphetamine (3 mg/kg), MDPV (1 mg/kg), or their corresponding vehicles. Approach latencies to enter the dark compartment during the training, before footshock, were evaluated. In the group given pre-training injection of propranolol or saline followed by a post-training administration of amphetamine or saline, RM ANOVA for approach latencies did not show significant effects of post-training treatment (F_(3,42)_ = 0.16, P = 0.92), context (F_(1,42)_ = 0.01, P = 0.91) or interaction between these two factors (F_(3,42)_ = 0.54, P *=* 0.66) (Table 2). In the group treated with a pre-training injection of propranolol or saline together with post-training administration of MDPV or saline, RM ANOVA for approach latencies indicated no significant effects of post-training treatment (F_(3,32)_ = 0.13, P = 0.94), context (F_(1,32)_ = 0.94, P *=* 0.34) or interaction between these two factors (F_(3,32)_ = 0.36, P *=* 0.79) (Table 2).

# Dopaminergic activation mediates the effects induced by amphetamine, but not MDPV, on memory generalization

Rats were treated with an intraperitoneal injection of the dopamine receptors antagonist cis-flupenthixol (0.25 mg/kg) or saline 30 min prior to training, followed by a post-training administration of the effective doses of amphetamine (3 mg/kg), MDPV (1 mg/kg), or their corresponding vehicles. Approach latencies to enter the dark compartment during the training, before footshock, were evaluated. In the group given pre-training injection of cis-flupenthixol or saline together with post-training administration of amphetamine or saline, RM ANOVA for approach latencies indicated no significant effects of post-training treatment (F(3,34) = 0.05, P = 0.99), context (F(1,34) = 0.003, P = 0.96) or interaction between these two factors (F(3,34) = 0.28, P = 0.84) (Table 3). In the group given pre-training injection of cis-flupenthixol or saline together with post-training administration of MDPV or saline, RM ANOVA for approach latencies did not indicate significant effects of post-training treatment (F(3,38) = 0.65, P = 0.59), context (F(1,38) = 0.07, P = 0.79) or interaction between these two factors (F(3,38) = 0.17, P = 0.92) (Table 3).

# Supplementary Tables

|  |  |  |  |  |  |  |  |
| --- | --- | --- | --- | --- | --- | --- | --- |
|  |  | | **Approach latencies in the Non-Shock box (s)** | | **Approach latencies in the Shock box (s)** | |  |
|  |  |  |  |  |  |  |  |
|  |  |  |  |  |  |  |  |
|  | saline  amphetamine 1 mg/kg amphetamine 3 mg/kg | | 14.6 ± 1.0  14.2 ± 1.4 13.4 ± 1.5 | | 14.5 ± 1.5  16.0 ± 0.9 15.8 ± 1.1 | |  |
|  |  |  |  |  |  |  |  |
|  |  |  |  |  |  |  |  |
|  | saline  MDPV 0.5 mg/kg MDPV 1 mg/kg ^-1^ | | 14.3 ± 2.1  14.5 ± 2.3 14.7 ± 3.4 | | 14.7 ± 1.3 12.7 ± 1.2 13.5 ± 2.7 | |  |
|  |  |  |  |  |  |  |  |
|  |  |  |  |  |  |  |  |
|  | Time spent to enter the dark compartment during the training (in seconds) of all groups. Data are expressed as mean ± SEM (n = 9-13 per group). | | | | | |  |
|  |  |  |  |  |  |  |  |
|  |  |  |  |  |  |  |  |

**Table 1 – Approach latencies in the Non-Shock and Shock boxes of rats post-training treated with amphetamine, MDPV or saline.**

|  |  |  |  |  |  |  |  |
| --- | --- | --- | --- | --- | --- | --- | --- |
|  |  | | **Approach latencies in the Non-Shock Box (s)** | | **Approach latencies in the Shock-Box (s)** | |  |
|  |  |  |  |  |  |  |  |
|  |  |  |  |  |  |  |  |
|  | saline-saline propranolol-saline saline-amphetamine propranolol-amphetamine | | 16.1 ± 2.1  14.1 ± 0.9 14.7 ± 1.1 15.4 ± 2.7 | | 13.6 ± 1.1 14.7 ± 1.6 16.9 ± 2.1 15.8 ± 3.2 | |  |
|  |  |  |  |  |  |  |  |
|  |  |  |  |  |  |  |  |
|  | saline-saline propranolol-saline  saline-MDPV  propranolol-MDPV | | 13.8 ± 2.1  13.9 ± 1.0  15.8 ± 3.4 14.0 ± 4.9 | | 13.9 ± 1.0 13.9 ± 1.3 12.3 ± 1.4 11.3 ± 1.7 | |  |
|  |  |  |  |  |  |  |  |
|  |  |  |  |  |  |  |  |
|  | Time spent to enter the dark compartment during the training (in seconds) of all groups. Data are expressed as mean ± SEM (n = 8-13 per group). | | | | | |  |
|  |  |  |  |  |  |  |  |
|  |  |  |  |  |  |  |  |

**Table 2 - Approach latencies in the Non-Shock and Shock boxes of rats treated with propranolol or saline 30 min prior to training together with amphetamine, MDPV or saline administered immediately after training.**

|  |  |  | |  |  |  |  |  |
| --- | --- | --- | --- | --- | --- | --- | --- | --- |
|  |  | | | **Approach latencies in the Non-Shock box (s)** | | **Approach latencies in the Shock box (s)** | |  |
|  |  |  |  |  |  |  |  |  |
|  |  |  |  |  |  |  |  |  |
|  | saline-saline cis-flupethixol-saline  saline-amphetamine  cis-flupethixol-amphetamine | | | 14.6 ± 2.9  13.8 ± 1.9 12.5 ± 1.6 15.8 ± 5.9 | | 13.9 ± 1.3 13.5 ± 3.0 15.1 ± 2.4 13.8 ± 1.8 | |  |
|  |  |  |  |  |  |  |  |  |
|  |  |  |  |  |  |  |  |  |
|  | saline-saline cis-flupethixol-saline  saline-MDPV  cis-flupethixol-MDPV | | | 11.8 ± 2.8  14.4 ± 3.7  12.4 ± 3.1 17.2 ± 5.1 | | 12.5 ± 1.5 13.5 ± 2.3 15.7 ± 3.0 16.6 ± 3.6 | |  |
|  |  |  |  |  |  |  |  |  |
|  |  |  |  |  |  |  |  |  |
|  | Time spent to enter the dark compartment during the training (in seconds) of all groups. Data are expressed as mean ± SEM (n = 8-11 per group). | | | | | | |  |
|  |  |  |  |  |  |  |  |  |
|  |  |  |  | |  |  |  |  |

**Table 3 - Approach latencies in the Non-Shock and Shock boxes of rats treated 30 min prior to training with cis-flupenthixol or saline together with amphetamine, MDPV or saline administered immediately after training.**
